# Implications of hypocobalaminemia as a negative prognostic marker in juvenile dogs with parvovirus enteritis

**DOI:** 10.3389/fvets.2024.1426664

**Published:** 2024-07-16

**Authors:** Nicole Luckschander-Zeller, Bettina Giani, Pavlos G. Doulidis, Hanna D. Plickert, Alexander Tichy, Rodrig Marculescu, Ilse Schwendenwein, Iwan A. Burgener

**Affiliations:** ^1^Department for Companion Animals and Horses, Division of Small Animal Internal Medicine, University of Veterinary Medicine of Vienna, Vienna, Austria; ^2^Tierklinik Parndorf, Parndorf, Austria; ^3^Department for Biomedical Sciences, University of Veterinary Medicine of Vienna, Vienna, Austria; ^4^Clinical Institute for Laboratory Medicine, Medical University of Vienna, Vienna, Austria; ^5^Central Laboratory, Department for Pathobiology, University of Veterinary Medicine of Vienna, Vienna, Austria

**Keywords:** cobalamin (CBL), parvoviral enteritis, canine, methylmalonic acid, outcome

## Abstract

**Introduction:**

Canine Parvovirus 2 (CPV-2) infection poses a significant global health risk to susceptible dogs. Hypocobalaminemia, defined as reduced serum cobalamin (CBL) concentrations, is a recognized complication in chronic enteropathies in adult dogs but remains poorly understood in the context of acute enteropathies, especially in young dogs. The aim of this study was to investigate the frequency and severity of hypocobalaminemia in young dogs with parvovirus enteritis and evaluation of CBL as a predictor of outcome.

**Materials and methods:**

Thirty client-owned dogs diagnosed with parvovirus infection and thirty healthy controls were enrolled. Clinical, hematological, and biochemical tests, including CBL and serum methylmalonic acid (MMA) concentrations, were assessed.

**Results:**

Results indicated a significantly higher prevalence of hypocobalaminemia in dogs with parvovirus enteritis compared to healthy controls, as well as a significant correlation with a disease severity score. Moreover, survivors demonstrated higher CBL concentrations than non-survivors, suggesting an eventual prognostic value of CBL status. However, parenteral CBL supplementation showed no significant effect on serum CBL or MMA concentrations, highlighting potential challenges in CBL uptake at the cellular level.

**Discussion:**

Hypocobalaminemia in this population is caused by multiple factors such as reduced nutritional absorption, gastrointestinal losses, and increased metabolic demands. Further research is needed to develop tailored management strategies, evaluate the effectiveness of CBL supplementation, and understand the mechanisms behind hypocobalaminemia in parvovirus infection.

## Introduction

Canine Parvovirus 2 (CPV-2) is a small, nonenveloped DNA-virus that spreads predominantly via fecal-oral or the oronasal route (1). First detected in the seventies of the last century, CPV-2 has continuously evolved into more virulent subspecies (CPV-2 a, b and c), representing a worldwide health risk to susceptible populations (2, 3). Clinical symptoms arise from cell death mediated by intracellular viral replication, predominantly affecting tissues with high cell turnover rates (enterocytes, cells of the bone marrow, thymic tissue, cardiac monocytes) (4–6).

Cobalamin (CBL) is a water-soluble cobalt containing B-vitamin that plays a pivotal role in cell metabolism and renewal, acting as a substrate in mitochondrial Krebs-cycle. Uptake of CBL features a complex multiple-step process, disruption of which might lead to hypocobalaminemia and consequently to CBL deficiency (7). Hypocobalaminaemia is defined as a serum CBL concentration between the lower range of quantification of the assay and the lower reference limit, while undetectable CBL concentration and an increased serum methylmalonic acid concentration are indicating a CBL deficiency (8). As carnivores and omnivores are not capable of producing significant amounts of CBL, most animals depend on CBL uptake via meat or eggs, as only a small amount can be produced by intestinal microbiota (9). Most commercial dog foods contain various amounts of supplemented CBL, thus additional supplementation is normally not required (10). Free CBL binds to R protein after being released from its bound form by gastric acid and activated pepsinogen in the stomach (11). The intrinsic factor (IF) produced mainly by the exocrine pancreas binds to CBL in the duodenum and the CBL-IF complex is absorbed in the ileum by receptor-mediated endocytosis (12–14). After separation of this complex, CBL binds to transcobalamin II which transports CBL to the tissues (8). In the target cells CBL plays a key role for enzymes like methylmalonyl-CoA mutase and methionine (15). A reduction of intracellular CBL concentration leads to a accumulation of methylmalonic acid (MMA) within the cell and consequently systemically. This promotes urinary excretion of MMA and urine acidification (8). Additionally the consequence of an impairment in the urea cycle caused by the MMA accumulation is an increase of the plasma ammonia concentration and among others, possible neurological complications and failure to thrive (8, 16–21).

Hypocobalaminemia and CBL deficiency is a well described complication in chronic enteropathies in adult dogs, due to a combination of malabsorption and dysbiosis (8, 22, 23), but data for serum CBL status and metabolism in dogs with acute enteropathies with a special emphasis on young dogs under one year of age is lacking. More specifically, two previous studies have evaluated CBL status in dogs with parvovirosis, but to our knowledge CBL status has not yet been evaluated as a predictor of outcome (24, 25). In accordance with human literature (26), suspected higher demands of CBL in young dogs with growth-related increased mitotic activity combined with CBL malabsorption due to parvoviral ileal enterocyte destruction and reduced dietary CBL intake, might predispose growing dogs with parvovirus enteritis to hypocobalaminemia (26). Preexisting or functional hypocobalaminemia at time of diagnosis might be a negative prognostic factor in dogs with parvoviral enteritis, as CBL has been shown to coordinate ileal epithelial and microbiota functions in mice infected with *Salmonella* spp. (27). Additionally, studies pertaining to hypocobalaminemic states in children describe physiologically higher CBL concentrations and requirements in growing individuals, especially in those younger than one year (28, 29).

Currently used reference intervals for CBL are derived from healthy adult dogs only, creating a potential risk for misjudging prevalence and severity of hypocobalaminemic states in growing pups, as young dogs seem to have different CBL demands and concentrations. CBL deficiency is reported with hematological and developmental disturbances, so that often irreversible peripheral and central neuropathies as well as immunodeficiencies in dogs and humans occur. Therefore, timely detection of hypocobalaminemic states and adequate supplementation of CBL is of high clinical relevance (18, 29–31). Reported CBL requirements in children prompt the necessity for development of age-related reference intervals for cobalamin and methylmalonic acid (MMA), a marker for adequate cellular uptake of CBL (28).

This study aims to fill this knowledge gap by determination of the frequency and severity of hypocobalaminemia in young dogs with parvovirus enteritis, as well as to contribute to the recognition and management of hypocobalaminemic states in growing dogs. Therefore we hypothesized that CBL will significantly correlate with disease severity and that patients that survive will demonstrate higher initial CBL concentration than non-survivors.

## Materials and methods

### Animals and study design

Ours was a single-center prospective observational clinical study. Client-owned puppies (less than 1 year of age) (*N* = 30) with acute onset of gastrointestinal signs (diarrhea, vomiting, anorexia) were presented to the Clinic for Small Animal Internal Medicine of the University of Veterinary Medicine Vienna, Austria. Included were those animals with confirmed parvovirosis. Diagnosis of parvovirus infection was made with the combination of consistent clinical signs and history and a positive result on fecal parvovirus antigen ELISA (IDEXX Parvo Snap^®^) or with a fecal PCR. After confirmation of parvoviral infection and clinical examination, the patients were hospitalized and transported to isolation wards allowing barrier nursing. Blood was collected via venipuncture for routine diagnostic workup (complete blood count and biochemistry panel) and determination of CBL and MMA concentrations at admission and after three to five days of hospitalization based on clinical requirements (time point 2). Clinical re-examinations and treatment were tailored to the individual patient’s needs. A routine fecal flotation was performed to rule out concomitant parasitosis if enough fecal material was available. All levels of clinical severity were included in this study. A diagnosis independent illness severity score (APPLE score) was applied upon admission and first examination to facilitate interindividual comparison within the study population. Patients with concurrent disease (e.g. intestinal parasites) or hematologic abnormalities unrelated to parvovirus infection were excluded from the study. Animals that had previously received CBL supplementation either parenteral or orally were also not considered.

Clinically healthy client-owned dogs (*N* = 30) with no history of recent diseases were considered for the control group. Dogs (age < 1 year old) of different breeds, body weight, and gender were included after fulfilling the inclusion criteria, including a detailed history, physical examination, and blood sampling performed by one of the authors (BG). Complete blood count (CBC), serum biochemical profile, and electrolyte measurements were performed. Only breeds other than Giant-Schnauzers, Shar-Peis, Beagles, Australian Shepherds and Border Collies were enrolled, as a genetic background for cobalamin deficiency has been confirmed or suspected (8). All control dogs had no history of gastrointestinal disease four weeks prior to presentation, were in excellent physical condition, judged healthy by the owner and no abnormalities were detected in physical examination. Any form of preceding CBL supplementation posed an exclusion criterion. Blood was collected by cephalic venipuncture to perform the same tests as done in the parvovirus group (PG) at admission day and a routine fecal parvovirus antigen ELISA (IDEXX Parvo Snap^®^) was performed to exclude CPV infection.

### Ethical approval

The study has been approved by the institutional ethics and animal welfare committee and the national authority according to Section 26ff. of Animal Experiments Act (Tierversuchsgesetz 2012—TVG 2012, BMBWF-68.205/0083-V/3b/2019). Written informed client consent was mandatory for study inclusion in both parvovirus and healthy group.

### Laboratory analysis

Complete blood count and a blood biochemistry panel were performed in a standard routine manner at the university’s central laboratory using the ADVIA 2120i (Siemens Healthcare Diagnostics GmbH, Austria) and Cobas c 501 (Roche Diagnostics, Austria). Cobalamin was measured in-house using a chemiluminescence immunoassay (Immulite 2000, vitamin B12, Siemens Healthcare Diagnostics Inc., Newark, Denmark) and the laboratory’s reference intervals (RI) for serum CBL (300–800 pg./mL; lower detection limit 150 pg./mL, upper detection limit 1,000 pg./mL) were used to determine cobalamin status. A dilution was not performed for values reported as over the upper detection limit. The upper detection limit value and the lower detection limit value were used to report values over and under the limit of detection, respectively. Serum supernatant was aspirated and stored frozen at −80°C for consecutive determination of serum methylmalonic acid (MMA) concentrations. Samples were shipped to the reference laboratory on dry ice for batch analysis and stored at −20°C until thawing at 37°C just before testing. MMA concentration was measured at the Clinical Institute for Laboratory Medicine, MedUni Vienna, Austria, using gas chromatography–mass spectrometry (MassChrom^®^, Chromsystems GmbH, Gräfelfing, Germany). CBL and MMA analysis costs were covered by the clinic.

### Medical management

The standard treatment protocol for parvovirus enteritis following initial stabilization included isotonic crystalloid fluid boli followed by continuous rate infusion (CRI) of crystalloids, supplemented with potassium-chloride (Kaliumchlorid„Fresenius “1 molar—infusion additive, Fresenius Kabi Austria GmbH, Graz, Austria) as needed and colloids if necessary (synthetic or natural). Antiemetic therapy with maropitant (1 mg/kg iv SID, Prevomax, Dechra Veterinary Products Germany GmbH, Germany), ondansetron (0.2–0.5 mg/kg iv BID—TID; Ondansetron B. Braun 2 mg/mL injectable solution, Melsungen, Germany) or metoclopramide CRI (1–2 mg/kg/d, Emeprid, Ceva Animal Health GmbH, Duesseldorf, Germany) was added tailored to the individual patient’s needs. Antibiotics were used and included amoxicillin/clavulanic acid (20 mg/kg iv TID; Curam, off-label use, Sandoz GmbH, Kundl, Austria) and Gentamicin (6.6 mg/kg iv SID, Gentavan, VANA GmbH, Vienna, Austria) after ensuring successful rehydration. Glucose was monitored closely, and hypoglycemia was treated with intravenous boli of glucose solution 50%, diluted 1:4 with crystalloids followed by CRI of 2.5–5% Glucose solution. In patients with more severe clinical manifestation and signs of systemic inflammatory response syndrome (SIRS), disseminated intravascular coagulation (DIC) or sepsis, fresh frozen plasma was given additionally to the above-mentioned treatment.

Enteral feeding was initiated as soon as possible via fixated nasogastric tube, which also allowed to remove excess stomach content to alleviate nausea and regurgitation before feeding. All dogs tolerated this procedure without requirement for sedation. For feeding purposes Royal Canin High Energy Liquid (1.5 kcal/mL) was used and the feeding amount and frequency were tailored to the patient’s needs, considering disease severity and gastrointestinal motility.

### Cobalamin supplementation

Hypocobalaminemia was defined as a serum CBL concentration <300 pg./mL, in accordance with the university’s laboratory reference intervals. Hypocobalaminemic dogs received parenteral cobalamin supplementation using a hydroxycobalamin formulation (Erycytol^®^ Depot 1 mg, G.L. Pharma Ges.m.b.H, Lannach, Austria) as a subcutaneous injection following the tested dosage and frequency recommendations of Texas A&M GI Laboratory^1^. CBL measurements were repeated after three to five days during routine blood work.

### Acute patient physiologic and laboratory evaluation score

To allow stratification of clinical illness and facilitate inter-patient comparison, all dogs in PG were subjected to a diagnosis independent illness severity score (32). Dogs in PG were scored using Acute patient physiologic and laboratory evaluation (APPLE)-score full and fast at admission day and on time point 2, synchronized to recheck of bloodwork.

### Statistical analysis

Statistical analysis was carried out at the Platform for Bioinformatics and Biostatistics, Department of Biomedical Sciences, University of Veterinary Medicine, Vienna, Austria, using the SPSS Software (IBM SPPS Statistics Version 28.0.1.0). Test for normality was performed using the Shapiro–Wilk test of normality. Normally distributed parameters were analyzed using ANOVA test with Bonferroni adjustment if needed, while not normally distributed parameters testing was performed either with Mann–Whitney U test or Kruskal–Wallis test for multiple comparisons. Correlations were evaluated using the Spearman’s rank correlation coefficient followed by regression analysis. A *p* value <0.05 was considered as statistically significant.

Estimation of the adjusted reference range of the 30 healthy dogs was performed with the parametric method by calculating the mean and standard deviation to determine the range of values that fall within the 95% confidence interval.

## Results

A total of thirty dogs (*N* = 30) were included in the PG. Median weight of the dogs in the PG upon admission was 3.55 kg (range 0.96–21.4 kg) and mean age was 2.6 months ± 0.67 months (22 days). Of these dogs, 63.3% (*N* = 19) survived to discharge and 36.7% (*N* = 11) died during disease. Mixed breeds comprised 33.3% (*N* = 10) of the group population, followed by Labrador Retrievers (20%, *N* = 6), Maltese dogs (10%, *N* = 3), Pomeranian (6.6% *N* = 2) and other breeds with 1 (3.3%) representative each (German Shepherd Dog, Husky, Toy Poodle, Jack Russel Terrier, Rottweiler, Cane Corso Italiano, Chihuahua, Gordon Setter, American Staffordshire Terrier). Eleven (36.7%) dogs were female and 19 (63.3%) were male.

Thirty (*N* = 30) dogs were enrolled as healthy controls (HC). The HC consisted of client owned dogs with mean age of 5.9 months (±2.9 months) and median weight of 18.20 kg (range 4.61–30.05 kg), of which 15 were intact males and 15 were intact females of the following breeds: 6 Australian Cattledogs (littermates), 1 Basset Hound, 5 Golden Retrievers, 12 Labrador Retrievers, 1 Labrador mix, 1 Samoyed, 1 Scotland Sheepdog, 1 Shetland Sheepdog, 1 Styrian Wire-Haired Bracke and 1 mixed breed dog.

Dogs in the PG had a significantly lower weight when compared to the HC dogs (median 3.55 kg, range 0.96–21.4 kg and median 18.20 kg (range 4.61–30.05 kg), *p* = <0.001). Mean APPLE score (fast) was 22.27 (±3.60) and mean APPLE score (full) was 18.21 (±6.21) in the PG upon admission. Mean hematocrit was significantly lower in the PG (34.72% ± 4.41%), when compared to the HC (39.5% ± 5.3%, *p* < 0.001) (Table 1). As expected, patients in the PG had significantly lower WBC, total neutrophil counts, total protein (TP), and albumin (ALB) concentrations when compared to HC (Table 1). No other statistical significance was found on comparison of the other hematological and biochemical parameters (all *p* > 0.05).

**Table 1:** Table of relevant hematological and biochemical parameters, that showed significant differences between dogs with parvovirus infection upon admission and healthy controls.

Test	PG (Mean ± SD)	HC (Mean ± SD)	*p* value
Hematocrit %	34.72 ± 4.41	39.5 ± 5.3	*p* < 0.001
WBC/μL	6,332 ± 4,293	11,960 ± 3,098	*p* < 0.001
Neutrophils/μL	4,347 ± 3,820	6,267 ± 1977	*p* = 0.018
Total Protein g/dL	4.59 ± 0.90	5.28 ± 0.61	*p* = 0.001
Albumin g/dL	1.9 ± 0.46	3.3 ± 0.31	*p* < 0.001

PG, parvovirus group; HC, healthy controls; SD, standard deviation; WBC, white blood cells.

From the 30 dogs in the PG, eighteen (*N* = 18, 60%) had CBL and MMA measurements at both time points and from them, eleven (*N* = 11, 36%) dogs, were hypocobalaminemic with serum CBL concentration below 300 pg./mL upon admission and received parenteral CBL supplementation. Of these dogs, 4 had MMA concentrations above the reference range. From the eleven dog that did not survive, a second measurement on time point 2 was possible in 9 dogs, as 2 dogs died before second sampling.

**Figure 1:**
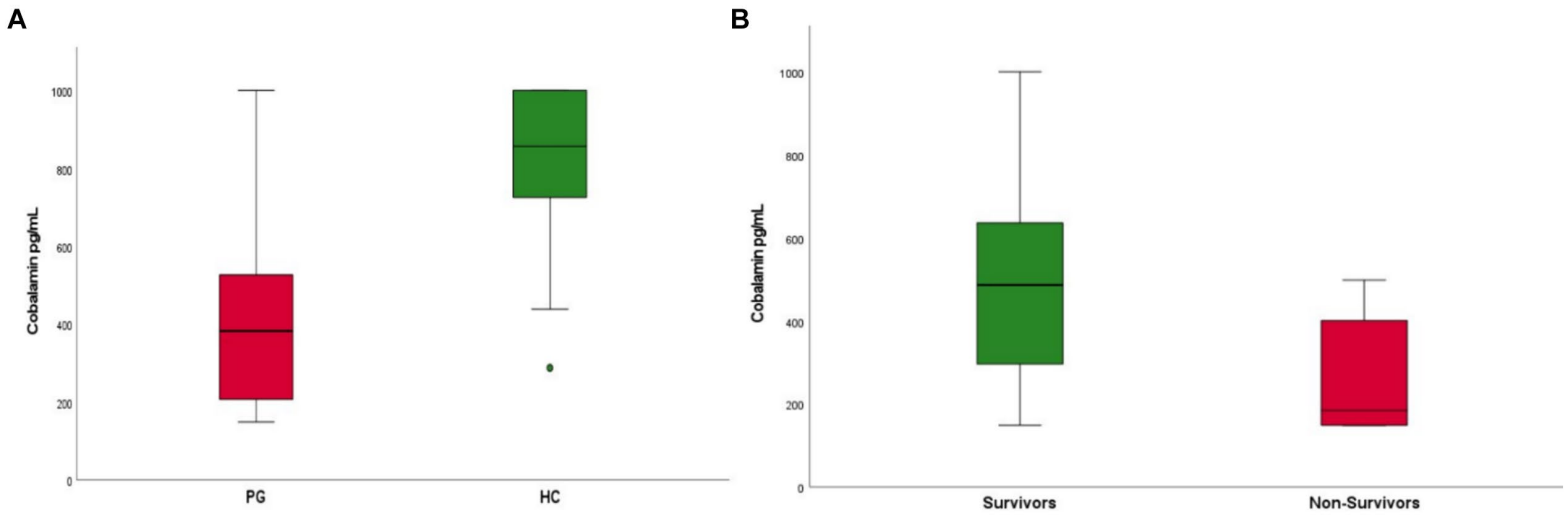
**(A)** Boxplot of cobalamin (CBL) concentrations in patients with parvovirus (PG) upon admission compared to healthy controls (HC). Patients in the parvovirus group showed lower CBL concentrations upon admission when compared to the healthy group (median 383 pg./mL, range 141–1,001 pg./mL and 857 pg./mL, 288–1,001 pg./mL respectively; *p* < 0.001). **(B)** Boxplot of CBL concentrations in patients that survived (survivors) compared to non-survivors. Survivors were found to have higher CBL concentrations upon admission compared to non-survivors [median 487 pg./mL (range 149–1001 pg./mL) and 185 pg./mL (range 149–499 pg./mL), respectively; *p* = 0.015].

Upon admission dogs in the PG showed significantly lower CBL concentrations compared to HC (median 383 pg./mL, range 141–1,001 pg./mL and 857 pg./mL, 288–1,011 pg./mL respectively; *p* < 0.001) (Figure 1A). In PG, higher CBL concentrations were detected in survivors compared to non-survivors [median 487 pg./mL (range 149–1,001 pg./mL) and 185 pg./mL (range 149–499 pg./mL), respectively; *p* = 0.015] (Figure 1B).

**Figure 2:**
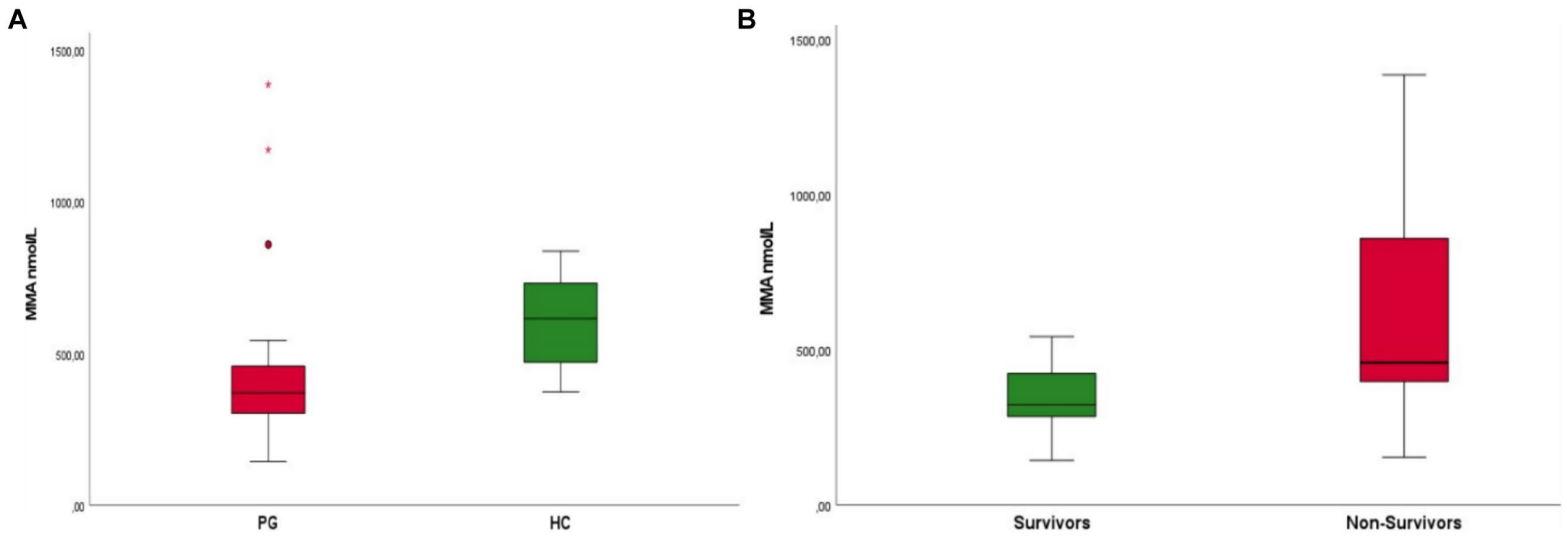
**(A)** Boxplot of patients with parvovirus (PG) upon admission compared to healthy controls (HC) Patients in the parvovirus group showed lower MMA concentrations upon admission when compared to the HC group (mean 452 nmol/L ± 287 nmol/L and 613 nmol/L ± 146 nmol/L, *p* < 0.001). **(B)** Boxplot of patients that survived (survivors) compared to non-survivors. Survivors were found to have lower MMA concentrations upon admission when compared to non-survivors [median 323 mmol/L (range 1,440–542 mmol/L) and 458 mmol/L (range 154–1385 mmol/L), respectively, *p* = 0.015].

Patients with parvovirus infection demonstrated significantly decreased MMA concentrations when compared to the HC group (mean 452 nmol/L ± 287 nmol/L and 613 nmol/L ± 146 nmol/L, *p* < 0.001) (Figure 2A). Survivors were also found to have lower MMA concentrations when compared to non-survivors [median 323 mmol/L (range 144–542 mmol/L) and 458 mmol/L (range 154–1385 mmol/L), respectively, *p* = 0.015] (Figure 2B).

Interestingly survivors had a slightly significant higher number of neutrophils in comparison to non-survivors (mean 3,328/μL ± 2,684/μL and 6,284/μL ± 4,964/μL, *p* = 0.046). No analysis of other hematologic or biochemical variables, including APPLE score (fast and full), showed statistically significant differences between survivors and non-survivors. A weak significant negative correlation between CBL and MMA was found in the HC (spearman’s rho −0.400, *p* = 0.035). The CBL concentrations in the HC exceeded those described for healthy adult dogs. Reference intervals derived from this group would be 706–1,001 pg./mL (mean 824.13 pg./mL +/− 194.9).

**Figure 3:**
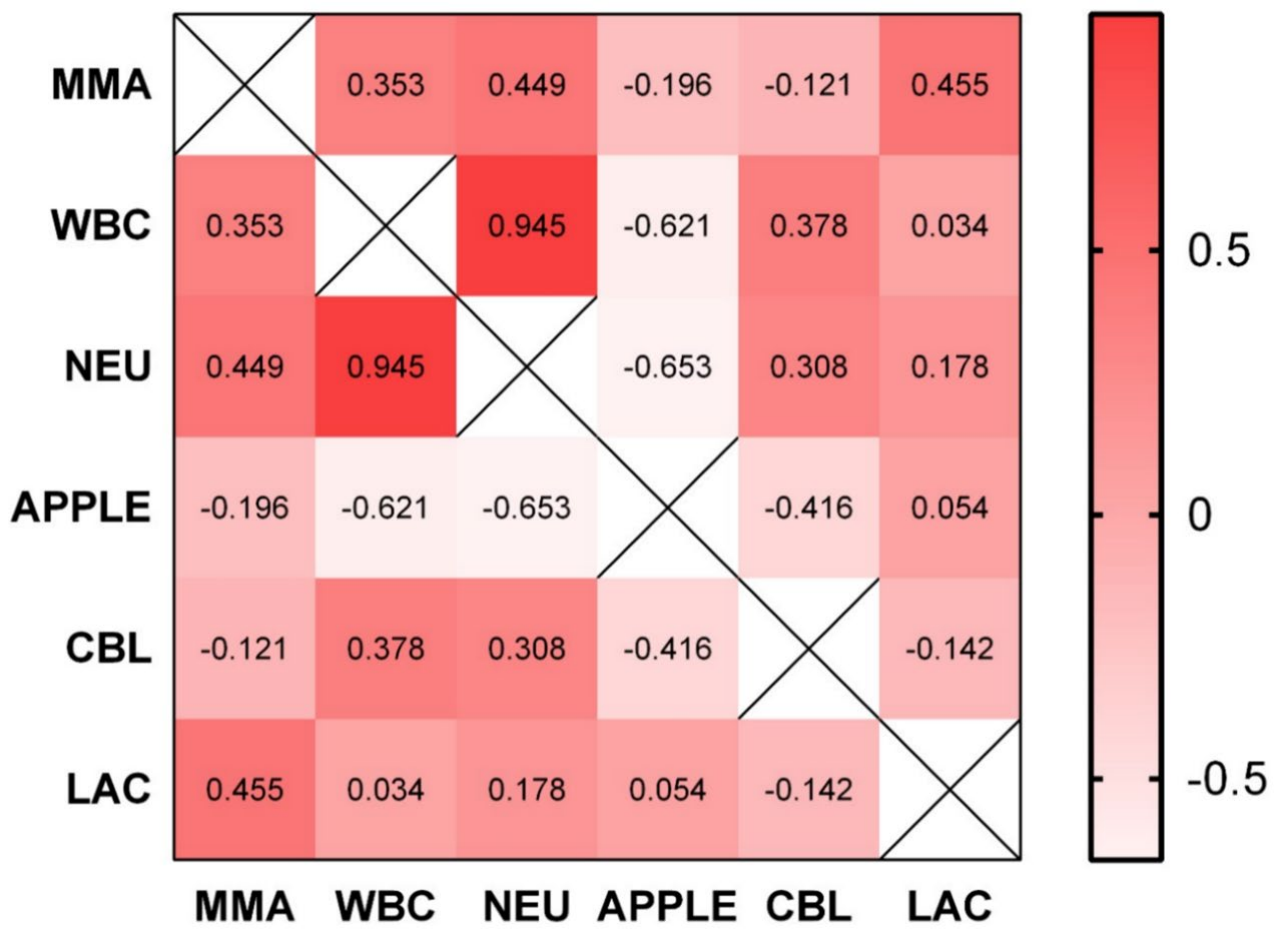
Correlation matrix illustrating the correlation between some of the evaluated parameters and biomarkers. No significant correlation between CBL and MMA was found. CBL and APPLE score are negatively correlated (spearman’s rho −0.416, *p* = 0.025). CBL was positively correlated with WBC count (spearman’s rho 0.378, *p* = 0.039). MMA positively correlated with lactate concentration (spearman’s rho 0.455, *p* = 0.022) and total neutrophil count (spearman’s rho 0.449, *p* = 0.024). MMA, methylmalonic acid; WBC, white blood cells; NEU, total neutrophils; APPLE, APPLE score; CBL, cobalamin; LAC, lactate.

In the PG we found no significant correlation between CBL and MMA at any given time point. In contrast, we detected a significant negative correlation between CBL and APPLE score (spearman’s rho −0.416, *p* = 0.025). Furthermore, CBL was weakly positively correlated with WBC concentration (spearman’s rho 0.378, *p* = 0.039), but not with total neutrophil concentration. Higher MMA concentrations positively correlated with lactate concentrations (spearman’s rho 0.455, *p* = 0.022) and total neutrophil count (spearman’s rho 0.449, *p* = 0.024). As expected, APPLE score upon admission was strongly negatively correlated to WBC (spearman’s rho −0.621, *p* < 0.001) and total neutrophil count (spearman’s rho −0.653, *p* < 0.001). All correlations are summarized in matrix format in Figure 3.

**Figure 4:**
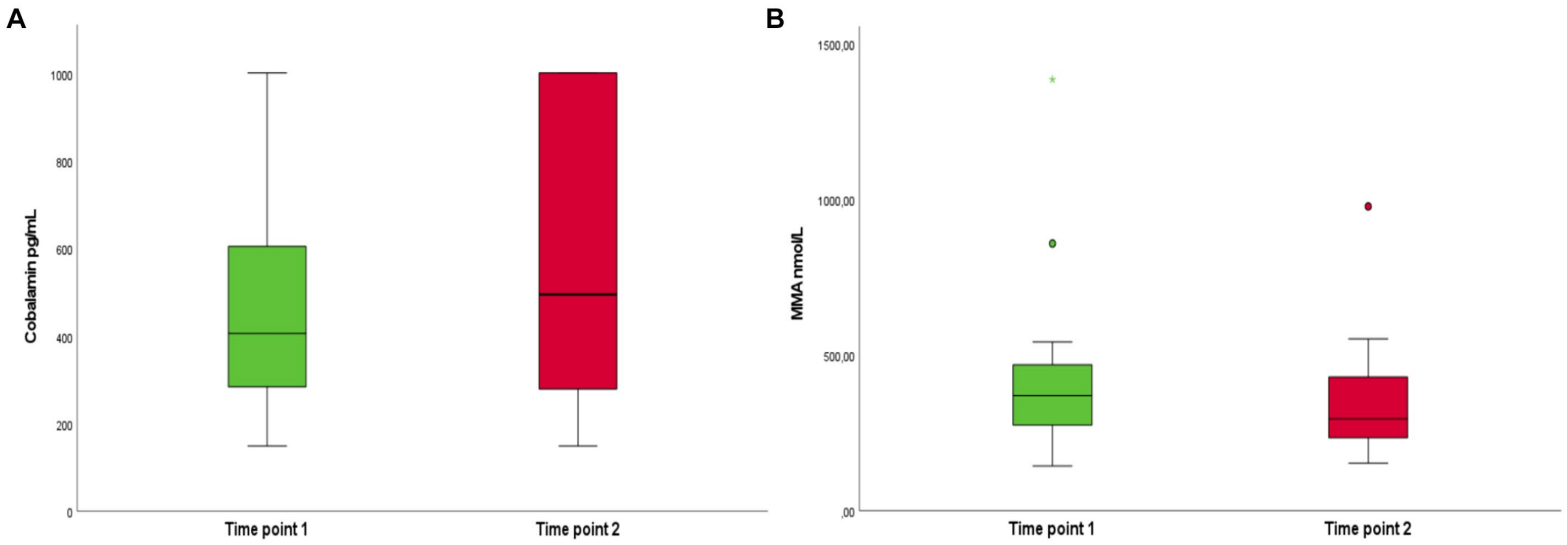
Boxplot of cobalamin (CBL) **(A)** and methylmalonic acid (MMA) **(B)** concentrations in patients with parvovirus upon admission (time point 1) and after 3–5 days (time point 2), after CBL supplementation. From the eighteen patients that had two measurements, eleven (*N* = 11, 36%) were hypocobalaminemic with serum CBL concentration below 300 pg./mL upon admission and received parenteral CBL supplementation. Nevertheless, parenteral supplementation of CBL had no statistically significant effect on CBL concentration and MMA (all *p* > 0.05).

In dogs with CBL concentrations above 300 pg./mL upon admission, mortality risk decreased by 50%, as most survivors demonstrated CBL concentration over this threshold. Results suggest a reduction of mortality risk by 40% if CBL concentration at time point 2 (3 to 5 days) exceeded 600 pg./mL. We detected no significant difference between CBL supplementation influence on outcome between day 3 (40% of dogs) or day 5 (60% of dogs). Parenteral supplementation of CBL had no statistically significant effect on CBL concentration and MMA (all *p* > 0.05), although an increase in CBL concentration was measurable in all animals receiving supplementation (median 505 pg./mL, range 105–1,001 pg./mL) (Figure 4). Of the 11 hypocobalaminemic patients that received CBL supplementation, eight had normal CBL concentration at time point 2, while 3 remained hypocobalaminemic.

## Discussion

In canine chronic enteropathies, hypocobalaminemia has been reported in 6–64% of the dogs (22, 33, 34). It has been shown that CBL supplementation in these patients can improve outcome (22, 35). The data presented in this study indicate an increased frequency of hypocobalaminemia in dogs under one year of age suffering from parvoviral enteritis. Indeed, as we hypothesized, serum CBL concentrations were significantly higher in patients that survived, when compared to non-survivors. Furthermore, serum CBL concentrations significantly correlated with other clinical or hematological parameters (negative correlation with APPLE score and weak positive correlation with total WBC count). In addition, evaluation of serum MMA concentrations and comparison between survivors and non-survivors, suggests a potential implication of intracellular CBL deficiency on outcome, as dogs that died had significantly higher serum MMA concentrations than survivors.

In healthy adults, hepatic cobalamin storage suffices the body for around 6 months. However, for some reason, this protective mechanism seems dysfunctional in our study population. Hypocobalaminemia in puppies with CPV-enteritis is most likely of multifactorial origin: unknown CBL status of the mother, possible reduced nutritional uptake, increased gastrointestinal loss, physiologically higher demands of growing individuals, immaturity of liver tissue such as for gluconeogenesis, impairment of cellular uptake and disturbance of recirculation mechanisms leading to storage depletion. As reference intervals for CBL derived from adult dogs were used to assess CBL status, the frequency and severity of hypocobalaminemia is probably underestimated in our population. Thus, the authors strongly recommend establishment of age-group-specific reference intervals for young dogs. In our study we found that the reference intervals derived from the HC group would be 706–1,001 pg./mL (mean 824.13 pg./mL +/− 194.9), although this result should be interpreted with caution as the minimum population number for safe reference interval estimation with the parametric method is 40 patients.

A recent study demonstrated that approximately 48% of dogs with acute gastrointestinal disorders have low CBL concentrations (36). Our results are consistent with previous studies, were young patients with parvoviral infection showed lower CBL compared to healthy controls (24). Interestingly though, in this study, the median serum CBL concentration in the healthy group was low (379 pg./L) and no difference was found between CBL concentration in young and adult dogs (24), suggesting in contrast to our results, that the reference interval is the same. Another study showed that dogs with parvoviral enteritis or other non-parvoviral acute enteropathy-patients have lower CBL concentrations when compared to healthy dogs, despite most of them were still within the reference interval (25). In this study serum MMA concentrations did not differ between groups, and there was no evaluation of outcome performed (25). However, our data indicates that low CBL concentration may be a negative predictor in young dogs with parvoviral enteritis, as outcome comparison among subgroups demonstrated higher CBL concentrations in survivors compared to non-survivors, while dogs with a CBL concentration under 300 pg./mL had 50% higher mortality risk. An obvious explanation for the decrease in CBL concentration in dogs with parvoviral enteritis is gastrointestinal loss due to the severe diarrhea and vomiting, in combination with decreased food intake. An additional possible explanation might be found in the fact that as suggested in humans, CBL status of children is highly dependent on maternal CBL status (28, 29, 37). Unfortunately, this information was not available in our study.

Another important consideration is that disruption of enterohepatic circulation might be a possible reason for premature depletion of stores, as an intact ileum is not only the sole location for effective, receptor mediated CBL absorption, but also a pivotal part of enterohepatic circulation. Cell regeneration after destruction by parvoviral replication causes higher requirements for CBL whilst at the same time enteral loss of important transport proteins further impairs CBL uptake. All this in combination with low CBL uptake due to inappetence and nausea illustrates the entire variety of possible mechanisms for hypocobalaminemia in our study population. Unfortunately, no long term follow up was possible, to determine if this is a persistent state or resolves with recovery.

Additionally, in our study we found significant differences in several hematological variables between groups (hematocrit, WBC, total neutrophils), but most importantly we detected a significant positive correlation between CBL and WBC (spearman’s rho 0.378, *p* = 0.039), which indicates that CBL concentrations are lower with worsening leukopenia and possibly disease severity. This hypothesis can be supported by the fact that we detected a significant negative correlation between CBL and APPLE score (spearman’s rho −0.416, *p* = 0.025), a connection that was not found in a previous study (36).

Significantly decreased hematocrit as well as TP and ALB concentrations are common findings in patients with parvovirosis and are of multifactorial origin (GI bleeding, decreased food intake, acute inflammation, diarrhea, vomiting, young age, growth). A potential role of CBL deficiency in the pathogenesis of anemia in our PG cannot be completely ruled out, although the relationship between CBL and anemia in dogs has been critically challenged (38), considering also the fact that puppies have lower hematocrit than adult dogs.

It has been suggested and is generally accepted, that CBL supplementation should be considered when serum CBL concentration is less than 400 ng/L or 295 pmol/L, as molecular disturbances can be expected at these concentrations, supported by the fact that some dogs with normal-low CBL have increased serum MMA (7, 8, 39–41). In our PG, supplementation of CBL showed no significant effect on MMA concentration between time point 1 and 2 compared to patients that were not substituted at all, although the interval of the two time points was short. Furthermore, MMA decreased from time point 1 to 2 independently from supplementation. In addition, we detected no significant correlation between CBL and MMA in the PG, results that align with previous literature and contradict the hypothesis of intracellular CBL deficiency (25). On the other hand, the negative correlation between CBL and MMA was confirmed in the HC. A continuous gastrointestinal loss of CBL or transcobalamin II and other transport proteins has been proposed as a possible explanation for the lack of correlation between CBL and MMA in disease state (25). Dogs with parvoviral infection frequently receive antibiotics to treat or prevent possible bacterial translocation, secondary to intestinal epithelial loss, exacerbated by the virus causing leukopenia and neutropenia. The prolonged use of antibiotics could additionally lead to other long-term effects, to worsening dysbiosis of the intestinal microbiome and consequently worsening hypocobalaminemia and failure to respond to supplementation (36, 42, 43). In our PG all patients had to receive antibiotics, but no significant differences were found when different antimicrobial protocols were compared. Parenteral supplementation of CBL did not have a significant effect on outcome, which in combination with the insignificant effect on the MMA concentrations, possibly indicate poor uptake of CBL on a cellular level. This might also be logically interconnectable to inconclusive findings regarding the missing correlation of MMA and CBL concentrations in PG. Further studies including measurement of transcobalamin II and homocysteine might elucidate potential causes of persistent hypocobalaminemia despite supplementation (8, 44–47).

In our study fatality rate was 36.7% as 11/30 dogs did not survive. This is a higher mortality than usually detected in our referral hospital and it might have several explanations. Many of the dogs that did not survive, were already presenting more severe clinical signs or advanced stages of the disease upon admission compared to our typical referral hospital population. This could be due to a variety of reasons including delays in seeking veterinary care and financial considerations. It is also possible that there were epidemiological variations such as different strains of CPV that could have influenced the mortality rate. Given the higher fatality rate observed, the above-mentioned factors should be considered during result interpretation. The elevated fatality rate might also suggest that our study population might represent a more severe spectrum of CPV cases, which provides valuable insights into the management and outcomes of critically ill CPV patients, but also potentially limiting their transferability to less severe cases commonly seen in other settings.

The following limitations of our study warrant consideration: although patients were prospectively enrolled, we could not ensure complete group homogeneity as per breed, age, and weight. Furthermore, because of the patients age and recent ownership status, we cannot completely rule out past medical interventions that could have affected CBL status. As the CBL concentration of the mothers were unknown, hypocobalaminemia in puppies because of CBL deficient bitches or insufficient uptake of mother milk cannot be ruled out. However, maternal CBL status was unknown also for the HC, suggesting this to be a minor limitation, as results in CBL concentration still differ significantly between groups. An additional limitation to our study, is the lack of dilution to detect specific values of CBL in patients with concentration over the upper detection limit. Lastly, another limiting factor of our study is that CBL supplementation recommendations are based on adult animals and CBL supplementation requirements may be higher in puppies and younger dogs, similarly to the higher reference range detected in our study.

Further studies should focus on the benefit of CBL supplementation on the outcome in patients with parvoviral infection and shed more light to the multifactorial pathogenesis of hypocobalaminemia in acute gastrointestinal diseases.

## Conclusion

Hypocobalaminemia and CBL deficiency in young dogs with parvoviral enteritis is frequent and should be recognized and therapeutically addressed. Measurement and if needed supplementation of CBL should be considered in young dogs with parvoviral infection. Low CBL concentrations can indicate a more severe course of disease and worse outcome, although supplementation did not seem to have a significant effect in our cohort. Determination of age-related reference intervals for CBL and MMA, as well as customized cut off levels for hypocobalaminemia is mandatory to avoid underestimation of this condition.

## Data availability statement

The raw data supporting the conclusions of this article will be made available by the authors, without undue reservation.

## Ethics statement

The animal studies were approved by institutional ethics and animal welfare committee of the Veterinary Medicine University Vienna, Austria and the national authority according to Section 26ff. of Animal Experiments Act. The studies were conducted in accordance with the local legislation and institutional requirements. Written informed consent was obtained from the owners for the participation of their animals in this study.

## Author contributions

NL-Z: Conceptualization, Data curation, Formal analysis, Funding acquisition, Investigation, Methodology, Project administration, Resources, Software, Supervision, Validation, Visualization, Writing – original draft, Writing – review & editing. BG: Conceptualization, Data curation, Investigation, Methodology, Validation, Writing – original draft, Writing – review & editing, Formal analysis. PD: Data curation, Formal analysis, Software, Validation, Writing – original draft, Writing – review & editing, Investigation, Methodology. HP: Conceptualization, Methodology, Writing – original draft, Writing – review & editing. AT: Formal analysis, Methodology, Writing – original draft, Writing – review & editing. RM: Data curation, Formal analysis, Methodology, Writing – original draft, Writing – review & editing. IS: Conceptualization, Visualization, Writing – original draft, Writing – review & editing. IB: Conceptualization, Funding acquisition, Methodology, Project administration, Resources, Supervision, Validation, Visualization, Writing – original draft, Writing – review & editing.

## Footnotes

^1^ https://vetmed.tamu.edu/gilab/research/cobalamin-information/
